# A qualitative exploration of food portion size practices and awareness of food portion size guidance in first-time parents of one- to two-year-olds living in the UK

**DOI:** 10.1186/s12889-023-16647-y

**Published:** 2023-09-13

**Authors:** Alice Porter, Rebecca Langford, Carolyn Summerbell, Laura Tinner, Ruth Kipping

**Affiliations:** 1 Population Health Sciences, Bristol Medical School, University of Bristol, Bristol, UK; 2 School for Public Health Research, NIHR, London, UK; 3 Department of Sport and Exercise Sciences, Durham University, Durham, UK

**Keywords:** Portion size, Preschool children, Guidance, Portioning practices, Qualitative

## Abstract

**Background:**

Food portion size guidance resources aimed at parents of young children in the UK are freely available from a number of credible sources. However, little is known about whether parents are aware of, and use, any of these resources to guide their food portioning practices.

**Objectives:**

We aimed to explore the food portion size practices used by first-time parents living in the UK when feeding their one- to two-year-old child, and their awareness of and views on six food portion size guidance resources.

**Methods:**

Participants were recruited via parent Facebook groups and online parent forums. Online 1–1 semi-structured interviews were conducted, during which parents were shown images of six food portion size guidance resources to facilitate discussion. Data was analysed in NVivo 11 using a Reflexive Thematic Analysis approach.

**Results:**

Of the 27 participants, most were women (*n* = 25), white (*n* = 18), and educated to first degree level or higher (*n* = 24). First-time parents mostly relied on their own judgement and *“instinct”* to portion foods, based on their learned experience of how much their child ate on a day-to-day basis. This experience was used alongside physical indicators of food portion size, such as the size of children’s dishware and food packaging. Most participants were unaware of any of the six food portion size guidance resources we showed them; only four had read any of the resources. Parents suggested they had previously sought advice about weaning from a range of sources (e.g. online, friends, community groups) but would be unlikely to seek out specific food portion size guidance. Parents suggested recommendations on food portion size should acknowledge and highlight parents’ perception that *“every child is different”*.

**Conclusions:**

Existing food portion size guidance resources for parents of young children in the UK are ineffective as they have poor reach and impact. We suggest parents should be involved in developing novel strategies to promote age-appropriate consumption and healthy weight gain in young children.

**Supplementary Information:**

The online version contains supplementary material available at 10.1186/s12889-023-16647-y.

## Background

Early childhood is a critical period for the development of child eating behaviours and food preferences [1]. Within the first year of life, children move from breastfeeding and/or bottle feeding to complementary feeding (where tastes and textures are introduced). From the age of one, children should be consuming a modified adult diet (often consisting of two to three meals and snacks per day) [2]. During this time, parents/carers have the greatest influence over what, when, where, and how much a child consumes [3].

Infants are born with an innate ability to self-regulate their energy intake, [4] which baby-led weaning during the complementary feeding stage can help to support [5]. However, as children grow and are exposed to external feeding cues, this ability can diminish, especially among children who are genetically susceptible to overeating [4]. The portion size of a served food/meal acts as an external cue to eat. Evidence suggests serving portion sizes to children which are larger than age-appropriate leads to excessive energy intake [6] and excessive weight gain during the preschool years [7].

Previous research suggests parents of preschool children (defined as one to five years) are interested in following portion size recommendations but are not generally aware of any available [8, 9]. Instead of following guidance, parents often report using instinct and experience to serve their children [10]. First-time parents of young children often seek information about feeding [11] and may be a particularly receptive audience to receiving and following portion size guidance. However, little is known about the portioning practices of first-time parents of young children, who have less previous experience of feeding to rely on. There is also sparse evidence about how UK portion size guidance are used among first-time parents.

A systematic grey literature review identified 22 online, freely available portion size guidance resources aimed at parents and childcare providers feeding preschool children in the UK and Ireland [12]. There may be a translational gap between the development of resources and the dissemination to parents/carers, which has not been previously explored. This qualitative study sought to understand the portioning practices used by first-time parents of one- to two-year-olds and explore parental awareness and views on six of the portion size guidance resources, previously identified and critically reviewed [12]. The six portion size guidance resources were selected because these were intended to be used by parents in the UK.

## Methods

### Participants

First-time primary caregivers of one- to two-year-olds living in the UK were recruited via study adverts posted in parent Facebook groups and online parent forums (e.g., Mumsnet) identified by the lead researcher (AP). Participants could reside anywhere in the UK. Participants were excluded if not a first-time parent/carer of a one- to two-year-old or had a child with a chronic condition or special feeding requirements because this could have influenced their knowledge of feeding guidance. First-time parents of one- to two-year-olds were selected because the study was interested in exploring portioning practices among parents with limited feeding experience. Participants ideally had access to a computer, laptop or tablet and the internet but phone interviews could be scheduled where access was not available. Purposive sampling was undertaken to recruit first-time parents from ethnic minority backgrounds.

### Study procedures

Participants who expressed an interest in the study were sent a participant information sheet, informational video, consent form and interview joining instructions. One-to-one semi-structured online interviews using Skype for Business were conducted with consented participants (COVID-19 restrictions prohibited face-to-face interviews). No phone interviews were conducted. Interviews took place between October 2020 and January 2021 and were conducted by AP. Interviews lasted between 40–90 min (mean = 58 min) and were audio-recorded using an encrypted device with participant consent. Following the interview, participants received a £20 shopping voucher as a thank-you.

The topic guide is presented in Additional file 1. Interviews explored parental portioning practices and views on six portion size guidance resources, identified in a previous grey literature review [12]. These six resources were selected because they were aimed at parents of preschool children in the UK. During the interview participants were shown a PowerPoint presentation of the six resources. This was used to facilitate discussion about their views on the resources. Table 1 presents the characteristics of the six portion size guidance resources, including links to the resources. Demographic information was collected at the end of the interview.

**Table 1:** Characteristics of portion size guidance resources shown to participants

Resource number	Resource name	Organisation	Presents meals or individual foods	Includes foods high in fat and sugar	Resource link
1	Eating well: Packed lunches for 1–4 year olds	First Steps Nutrition Trust	Meals	No	Packed_lunches_Dec17.pdf (squarespace.com)
2	Portion sizes for toddlers	Infant & Toddler Forum	Individual foods	Yes	Portion Sizes for Toddlers—Infant & Toddler Forum (infantandtoddlerforum.org)
3	Every Baby Matters	NHS Bradford	Individual foods	Yes	Every Baby Matters Nutrition Guidelines—1 to 5-year-olds (bradford.gov.uk)
4	5532 a-day	British Nutrition Foundation	Individual food	No	5532-booklet-sept21.pdf (nutrition.org.uk)
5	Recipe and meal ideas	Start4Life	Meals	No	Weaning Recipes & Meal Ideas | Start for Life (www.nhs.uk)
6	Food portion book for 1–4 year olds	Bristol City Council	Individual foods	No	Food-Portions-Book-1–4-years.pdf (bristolearlyyears.org.uk)

Ethical approval was obtained from the Faculty of Health Sciences Research Ethics Committee, University of Bristol, UK. The Standards for Reporting Qualitative Research (STOR) were followed (Additional file 2) [13].

### Data analysis 

Interviews were transcribed verbatim and anonymised. Transcripts were analysed using a reflexive thematic approach [14, 15] with NVivo used for data management and retrieval. This flexible, iterative analytic approach involved familiarisation with the data, coding, generating initial themes, reviewing themes, defining and naming themes, and writing up. A combination of inductive and deductive coding was used. Initial coding was conducted independently by three researchers (AP, RK, LT) to provide a range of expertise and perspectives (AP coded all, LT coded 5, RK coded 2). Initial codes were discussed, which informed the development of the coding framework, which was then applied to the remaining transcripts by AP. The coding framework was regularly discussed and updated. Themes and sub-themes were developed, discussed, refined and named.

### Reflexivity 

This study was approached from a constructionist epistemology (considering language to be an important aspect of how experience is communicated) and experiential orientation (appreciating participants’ responses reflect their own personal state) [16]. Data collection and analysis was influenced by the researchers’ social context, and experiences (researchers were White, highly educated females, with or without children, all with an interest in child health) and theoretical positioning.

## Results

### Participant demographics

Twenty-seven first-time parents of one- to two-year-olds were recruited: 25 mothers and two fathers. Table 2 presents the participant demographics. Most participants were White (*n* = 18 [67%]) and aged between 31 and 35 years (*n* = 17 [63%]). Twenty-four (89%) participants had a first degree or higher and 26 (96%) were married or living with a partner. Child age ranged between 13 and 24 months.

**Table 2:** Participant demographic characteristics (*N* = 27)

Sample characteristics		N	%
**Age**	25–30	4	15
	31–35	17	63
	36–40	4	15
	41–45	1	4
**Gender**	Female	25	93
	Male	2	7
**Ethnicity**	White British	18	67
	Bangladeshi	4	15
	Pakistani	1	4
	Black Caribbean	1	4
	Chinese	1	4
	White Other	1	4
	Mixed	1	4
**Region of UK**	London	2	7
	East of England	2	7
	West Midlands	2	7
	Yorkshire and Humber	1	4
	North West	3	11
	South West	15	56
	South East	1	4
	Wales	1	4
**Education**	A levels/NVQ/GNVQ	3	11
	First degree or equivalent	18	67
	Higher degree or equivalent	6	22
**Employment status**	Full time	10	37
	Part time	12	44
	Stay at home parent	3	11
	Unemployed	1	4
**Marital status**	Married	19	70
	Living with partner	7	26
	Single	1	4
**How participants found out about study**	Facebook	12	44
	Through a friend or relative	14	52
	Online parent forum	1	4
		**Mean (SD)**	**Range**
**Age of child (months)**		18 (3)	13–24

*Abbreviations: NVQ* National Vocational Qualification, *GNVQ* General National Vocational Qualification

### Portioning practices of first-time parents

#### Parental experience and learning

When first asked, many parents referred to using their *“instinct”* or just *“winging it”* when serving portions to their child. However, when probed further, they described learning over time from observing the amount their child ate (and wasted) on a meal-to-meal basis, using this as a reference when serving portions at the next meal. Although this sample of parents had limited feeding experience (being first-time parents of one- to two-year-olds), parents suggested they had gained confidence over time from past feeding experiences, which they now used to decide portions intuitively through *“eyeballing”* and guessing approximate amounts. This contrasted with experiences during weaning, where parents tended to measure foods more frequently. Although most parents did not know the official portion size recommendations for one- to two-year-olds before being shown the resources, parents felt confident to serve their child appropriate portions across a range of meals and foods, and to allow their child autonomy to dictate how much they ate (within limits and depending on perceived healthiness of the food).

*“When we were first getting onto proper food, he was maybe getting too much and then he would leave it because he wasn’t eating it so I think you just get used to how much [food] they get through” (Participant 4-Parent of 19-24-month-old)*

*“You listen to all the advice and then you realise that actually your own instincts are better.” (Participant 18-Parent of 19-24-month-old)*

#### Physical indicators 

First-time parents also used physical indicators to determine portion sizes. Parents were often guided by the size of children’s dishware, believing the amount that fits in a child-specific bowl, plate or Tupperware was a child-appropriate portion size. Similarly with packaged foods specifically marketed for young children (such as *Organix™, Ella’s Kitchen™* and *Kiddylicious™*), parents served the whole packet because they felt this was an age-appropriate portion size.

*“I think because I’ve got his little kiddie bowl and the plate, I just put it in that and then I kind of know obviously if it’s a bowl for a kid then I suppose that should be the right portion size.” (Participant 8-Parent of 19-24-month-old)*

Parental judgement alongside physical indicators was often used. For example, a whole packet might not be served if the food was perceived to be unhealthy and a full bowl of one single food was sometimes perceived to be too much (e.g. a full bowl of yoghurt). A few parents suggested their child became overwhelmed when presented with a large, full plate of food. In this instance, parents served less on the plate to start, followed by a second serving.

*“I’ve just got my idea about what [her] size portion would be for all different types of food. So, with yoghurt it would probably only be about half full.” (Participant 2-Parent of 19-24-month-old)*

*“Giving her a little bit to look at, think about, sort of process and then eat – that is the best way to do it rather than pile the whole portion in front of her… I think it’s just about, just not being overwhelmed by a big mass of food in front of her” (Participant 7-Parent of 12-18-month-old)*

A few parents used their own portion sizes to help determine the portion size for their child and whether to offer additional servings. A portion that was *“smaller than*” or *“less than half”* of their adult portion was deemed appropriate.

*“With porridge, she has quite a big bowl in the morning… I won’t give her more because if she has any more, it’s bordering on an adult’s portion” (Participant 12-Parent of 19-24-month-old)*

#### Awareness and use of existing portion size guidance for feeding preschool children

Participants were shown the six portion size guidance resources and asked about recognition. Resource five (Start4Life) was the most recognised resource, recognised by seven parents (26%). However, parents referred to this resource as a source of information for weaning to solid foods rather than something used now. Resource one (First Steps Nutrition Trust) was recognised by two parents, Resource three (National Health Service (NHS) Bradford) by two, Resource four (British Nutrition Foundation) by one and Resource six (Bristol City Council) by two. No parents recognised Resource two (Infant & Toddler Forum).

Only four of the 27 (15%) first-time parents had read any of the six portion size guidance resources. Three of these parents had found the resources useful, however one parent said *“I’ve read it, but I didn’t really tend to take much notice of it” (Participant 21-Parent of 12–18-month-old).* Parents described obtaining feeding advice and guidance more generally (e.g. to obtain information about how to wean, foods to avoid, how to manage certain feeding situations such as when a child is not eating) from a range of sources, including the internet, family and friends, social media, books, health professionals (e.g. health visitors), and community groups (e.g. children’s centres). In addition, the NHS website was often mentioned as a trustworthy information source for official guidance. However, very few parents described using these sources to obtain information about how much to feed their child specifically.

Several parents explained they had read a lot during the weaning stage and so needed less guidance now. For some parents, seeing these resources acted as a *“reminder”* to continue seeking information during the preschool years. However, the resources were viewed as a *“guide”* or *“reference”* to aid decision making and *“give you an idea of what’s appropriate"* rather than something which should be rigorously followed. Conversely, other parents deemed the resources unnecessary because they were confident in feeding their child the right amount, had an established feeding routine, and were happy being led by their child’s hunger cues rather than the guidance.

*“I definitely used to look at things much more around that weaning age… It’s probably not a bad idea to just go and have a little look at typical portion sizes, and just try and keep up to date with her age group a little bit.” (Participant 16-Parent of 12-18-month-old)*

*“It was the first year where I was really concerned and really worried and I wanted to do it according to the guidance. But now, you say after one year maybe, you will get knowledge. You get experience [of] how much he wants to [eat]” (Participant 25-Parent of 12-18-month-old)*

In addition, some parents mentioned time was a barrier due to going back to work, so they no longer had time to read online information. At this stage, parents were more likely to look online for meal inspiration than for specific guidance or advice.

*“I’ve sort of got into a routine with it now and got more confident and I guess being back at work I’ve not had as much time to be looking.” (Participant 12-Parent of 19-24-month-old)*

*“Quick meal ideas, that’s all I ever want really.” (Participant 6-Parent of 19-24-month-old)*

#### Content, structure and accessibility of the portion size guidance resources 

Resources two, three, four and six presented individual food portion sizes, while resources one and five presented meal portion sizes (see Table 1). For example, this webpage provides an example of the British Nutrition Foundation resource presenting individual food portion sizes: 5532-booklet-sept21.pdf (nutrition.org.uk) [17] and this webpage provides an example of the NHS Start4Life resource presenting meal portion sizes: Beefy veg curry—Weaning recipes—Start for Life—NHS (www.nhs.uk) [18]. Some parents liked resources which showed portion sizes for specific foods (e.g. portions of pasta or vegetables) and how these foods could make up a balanced diet. Other parents preferred resources which showed actual meals (e.g. curry) because they showed how foods could be combined and offered meal inspiration. Although one Bangladeshi parent felt the resources only recommended “Westernised” meals, which they would not cook at home.

*“[it] is really handy knowing what amount of each food group is needed and then like I said, once you see that it sticks with your mind, you get used to cooking it and you then just know how much of what you need to keep giving them” (Participant 8-Parent of 19-24-month-old)*

“*I’d probably look at more websites, pages that have something that can feed the parents as well. It’ll say ‘portion size for two adults and a child’, rather than – I don’t really make individual portions like that now.” (Participant 12-Parent of 19-24-month-old)*

*“I find a lot of the meals that are there on-line are sort of Westernised if that makes sense. There’s not much for sort of people in our ethnic minority with the sort of ingredients we have in our cupboards.” (Parent of 12-18-month-old/P22)*

Only resources two and three presented recommended portion size limits for foods high in fat and sugar; the other resources either did not mention these foods or more generally suggested avoiding them. Most parents thought it was appropriate to include these recommended portion size limits to remind parents which foods should be restricted and by how much. Although many parents were currently restricting these foods from their child’s diet, they acknowledged these foods would probably *“creep into their lives at some point”* and having guidance was helpful. Conversely, a couple parents felt strongly that resources should not include unhealthy foods because this might “*signal that it’s okay to give them*” and instead should provide healthy ‘treat’ options.

*“It’s useful to know how much of it they have or what you should be aiming for and when they’re reaching for their fifth biscuit, perhaps half a biscuit is enough, so yeah, I like resource two.” (Participant 6-Parent of 19-24-month-old)*

Showing parents the resources sparked discussion about the resources’ visual appeal, length, accessibility and usability. Some parents were interested in reading the resources and found them *“interesting”, “short [and] sharp”*, whereas others were uninspired, finding them *“busy”* and *“not simple”*. Parents preferred the short, concise and visual resources. Parents liked the presentation of portion size recommendations in simple concise tables, which could be screenshotted or downloaded to their phones. Parents were more drawn to the resources with bold colours, a visual front cover and images of foods and meals, which could help them to better gauge how big the portion sizes were.

*“I think that’s really good to have visually, to kind of work out [portion size], actually, yes, otherwise I think it would be really easy to just give them what you give yourself.” (Participant 10-Parent of 12-18-month-old)*

#### Portions consumed compared to the guidance 

During the interviews, many parents compared the portions their child consumed to the recommended portions in the resources. For some parents, the comparison provided reassurance their child was eating enough for their age. For others, parents became anxious their child wasn’t eating enough.

*“That little resource thing, I think if I had that in my arsenal, I’d be like, ‘It’s all right! I’m doing all right!’…It actually makes me feel better, so if he ever does not eat it all, then I’m like, ‘Well, he’s having more than he should have anyway, so it’s fine.’” (Participant 17-Parent of 12-18-month-old).*

*“I think following something like that probably wouldn’t be ideal because not every child would stick to something like that and it’d just end up making me more anxious.” (Participant 22-Parent of 19-24-month-old)*

In contrast, parents did not seem particularly concerned if their child was consuming more than recommended. Only a few parents suggested they might *“cut down on certain foods”* (especially ‘unhealthy’ foods) if their child was consuming more than recommended, to avoid overeating and excessive weight gain.

The notion that *“every child is different”* seemed to ring true for many parents. Some parents felt the guidance did not reflect this, nor did it reflect the meal-to-meal and day-to-day variation in how much their child ate. Parents wanted to be led by their child’s appetite and hunger and respond to those needs appropriately over the course of the day. One parent suggested guidance resources should reflect that all children eat differently and should instead inform parents about the contexts in which portion sizes may be of concern.

*“I’m sure sometimes she has eaten more than a tablespoon of rice or a tablespoon of mashed potato in one sitting. I wouldn’t be worried about that because sometimes like in a day, some of her meals and snacks, she’ll hardly eat anything and she’ll just pick at and then one or two of those meals, or snacks, she’ll seem to eat absolutely tons and I’m sure it’s just making up for the fact that she’s not really eaten anything all day.” (Participant 2-Parent of 19-24-month-old)*

## Discussion

Our study found that first-time parents were mostly unaware of and did not use any of the six portion size guidance resources aimed at parents feeding preschool children in the UK. Our findings suggest existing portion size guidance resources in the UK are ineffective as they have poor reach and impact on parental portioning practices. Although our sample consisted of first-time parents (and thus with limited feeding experience), we found by the time a child is one- to two-years-old, parents have gained enough experience to serve what they perceive to be appropriate portions for their child and prefer to be led by their child’s hunger, appetite and preferences (albeit within reason). Through ‘eyeballing’, estimation and observing how much their child eats and wastes on a meal-to-meal and day-to-day basis, parents serve portions similar to the amount last consumed by their child.

Parents of preschool children in previous studies have similarly been unaware of any portion size guidance [8, 10, 19, 20]. Tang et al., asked parents about their awareness of the UK Change4Life ‘me-sized meals’ campaign and found only 24% were aware of it [21]. In addition, those who were aware of the campaign had not considered using it to help decide portions [21]. Although parents of preschool children express an interest in following recommendations, [8, 9, 19] it is clear parents are not engaged with the guidance as it is currently presented.

Like parents in other studies, [8, 9, 19] the first-time parents in our study used physical indicators of portion size (e.g. dishware, package size, adult portions), alongside their own judgement and experience. In addition, a review of qualitative studies, Kairey et al., similarly identified ‘onus of control over portion size’ as a theme, [10] suggesting parents want to allow their child to self-regulate their intake while also applying some limits. This portioning practice is similar in nature to baby-led weaning [5] and suggests parents may want to continue to foster this feeding practice throughout the early years.

Due to the perceived prescriptive nature of the guidance, parents were reluctant to follow it because they felt the recommendations did not promote self-regulation, nor consider child individuality and the variability in children’s eating (between meals and days). Quantitative evidence suggests portion sizes do vary within preschool children, dependent on the eating context [22] and therefore it may be important for guidance to reflect this. First-time parents seemed more concerned with getting their child to eat enough over the course of a whole day than they were with the portions of individual foods or meals. Parents also seemed less concerned about their child eating more than the recommended amounts. Other studies similarly suggest parents are more concerned about providing a balanced diet for growth than portion sizes being too large [10, 23]. Prospective evidence suggests large meal size is a critical driver of excess weight gain during the preschool years [7]. Although the goal of public health researchers and paediatric health professionals may be to prevent excessive weight gain in children (in light of the current childhood obesity rates [24]), this may not be as important to parents at this age.

### Implications for practice, policy and future research 

Timing may be key to educate parents about age-appropriate food consumption during the preschool years and the importance of healthy weight gain. First-time parents’ feeding decisions during breast/bottle feeding and weaning are influenced by written materials, family and friends, health professionals and social media [11]. Many first-time parents in our study said they had searched for and followed online guidance during the weaning stage when they lacked experience and had more time (before returning to work), however were later more likely to only search for meal inspiration. To engage and educate parents at the opportune time, health professionals such as maternal child health practitioners and health visitors could provide parents with simple, concise, visually appealing, accessible (e.g. downloadable or able to screenshot) and reputable (e.g. NHS) resources during weaning (with a reminder to engage post-weaning). This would also require practitioners to receive regular training and updates about such resources. Development of infant feeding apps may promote parental engagement with guidance, [25] however future research with parents from a range of socioeconomic and ethnic backgrounds is required to establish the best methods of dissemination (e.g. health professionals, apps, children’s centres, peers, social media).

In terms of policy, increased regulation of those responsible for food (and dishware) manufacturing, supplying and advertising to families and young children may be required, as education and guidance alone are unlikely to have a large impact, and could increase existing inequalities [26]. Day et al., conducted a survey exploring parental perceptions and perceived impact of the UK Change4Life 100 kcal snack campaign (a campaign to encourage parents to provide lower calories snacks to their children) [27]. Parents found the campaign informative and memorable but there was little evidence of behaviour change [27]. Parents in this study and others [8, 10, 21] describe using dishware and food packaging to help guide their portioning. However, with so many child-targeted foods and dishware on the market, it is unknown whether these products are indeed suitable for preschool children. Action on Sugar reported 37% of child-targeted sweet snacks in the UK are high in sugars (with only 8% classified as low in sugar). Kiddylicious*™* and Organix*™* (mentioned frequently by parents in this study) contained among the highest amounts of sugar [28]. Policies to encourage or mandate food and dishware companies to reduce sizes, reformulate child-targeted products (especially those high in sugar) and ensure affordability of healthy options for all, such as introducing food manufacturer targets based on dietary guidelines and levies (taxes, fees or fines), or parent vouchers for healthy foods could provide effective environmental level strategies to promote age-appropriate food consumption [29–33].

Parents in this and other studies [10] suggest encouraging self-regulation is a key feeding practice they want to foster, which may be protective against excessive weight gain [7, 34]. However, further prospective or trial evidence is required to establish the associations between the portioning practices (identified in the qualitative literature [10]) and child weight gain, also considering how child appetite traits are likely to influence parental feeding practices and child consumption [35]. In addition, evaluating the impact of following portion size recommendations on child growth and weight is important to inform future use and adaptation of the existing resources.

To produce evidence-based but also reputable and appealing child feeding guidance, resources should be co-designed with parents, public health organisations and charities responsible for publishing guidance, health professionals responsible for disseminating guidance and public health researchers. Importantly, parents from ethnic minority and deprived backgrounds should be engaged to ensure appropriate content. For example, guidance should consider the types of foods presented to ensure cultural inclusion [36, 37] and affordability, especially in light of the current cost-of-living crisis [38].

### Strengths and limitations 

This study was the first to use a pragmatic method of showing primary caregivers portion size guidance resources to facilitate in depth discussions about their reach, appeal and usefulness. It was also the first to explore portioning practices among a sample of first-time parents. However, our study has limitations that should be acknowledged. Due to COVID-19 restrictions, interviews were conducted online, rather than face-to-face as originally planned. Though there were some distractions during interviews (e.g. technical issues or parents responding to their child), online interviews were largely advantageous because the researcher was able to recruit from across the UK, participants could do the interview in the comfort of their own homes and there was greater flexibility around scheduling interview times. However, individuals without access to IT may have felt less inclined to take part. In addition, online recruitment made it harder for the researchers to reach parents from deprived backgrounds. The sample included mostly highly educated mothers (only two fathers participated). Parents with lower levels of education may be even less likely to access child feeding resources, [11] therefore awareness of resources is unlikely to be higher than identified in this study. Although we used purposive sampling and one parent highlighted that the Western-centric nature of resources may not be appropriate for all ethnic backgrounds and cultures, the limited number of parents from ethnic minority backgrounds in our sample (*N* = 9) makes it difficult to draw strong conclusions about their utility for different population groups. Further community-based work is required to recruit and involve parents from deprived and ethnic minority backgrounds in research that aims to improve child health, to help reduce health inequalities. Data was not collected on parent or child BMI and therefore it is unknown whether portioning practices or use of guidance differs dependent on child or parent weight.

## Conclusions

This study suggests there is a translational gap between the dissemination and usage of portion size guidance resources in the UK, with poor reach and limited impact on awareness and practices of parents of one- to two-year-olds. First-time parents seek weaning advice but by the time their child is one year, most are confident to serve portions to their child based on experience of previous feeding occasions and do not seek portion size guidance. Involving parents in the development of novel strategies to disseminate, engage and promote child-appropriate portion size recommendations, which consider their current portioning practices (such as using child dishware, serving packaged foods, and being responsive to their children) are necessary. But so too, are other environmental policy responses to facilitate appropriate portion size servings, which do not depend on awareness and use of portion size guidance.

### Supplementary Information

**Additional file 1. **Topic guide.

**Additional file 2.** Standards for reporting qualitative research (STOR).

## Data Availability

The data presented in this study are available on reasonable request from the corresponding author.
