# Supplementary material for: A qualitative exploration of food portion size practices and awareness of food portion size guidance in first-time parents of one- to two-year-olds living in the UK
Source: BMC Public Health. 2023 Sep 13;23:1779. doi: 10.1186/s12889-023-16647-y (PMC10500748; doi:10.1186/s12889-023-16647-y)
Supplement: Supplementary file 1 — Additional file 1. Topic guide. [file 12889_2023_16647_MOESM1_ESM.docx]

Additional file 1. Topic Guide

| **Background info** |
| --- |
| Tell me a bit about you and your little one. What’s it like being a first-time parent? |
| What age did you start feeding him/her solid foods? |
| Briefly, what does he/she eat now in a typical day? |
| **The typical feeding experience** |
| Talk me through a typical meal with your child |
| Are you primarily responsible for feeding him/her? |
| Is your child involved in choosing what or how much food to eat? |
| Does how much your child eats vary from meal to meal or day to day? |
| **Concerns around portion size and feeding** |
| What kind of appetite does your child have? |
| Is how much your child eats a concern for you? |
| Are there any other concerns that you have when it comes to his/her eating? |
| **Decisions on portion sizes** |
| How do you decide how much to serve your child or how much or child eats? |
| Is there anything that influences your decision? |
| Do you feel that you have a good idea about what’s an appropriate amount for your child to be eating? |
| **Restriction of portion sizes** |
| Are there certain foods that you control portions of or restrict all together? |
| Are there certain foods that you don’t restrict portion of? |
| **Portion sizes specific to types of meals and foods** |
| Are there any foods that you find difficult to decide the portion of or to portion out? |
| How much would you decide to give your child of a pre-packaged food? |
| You say you decide how much to feed your child by… does this differ if it’s a meal or a snack? |
| What do you do if you are eating out? |
| **Who influences portion size decisions** |
| Are there other people involved in deciding how much to feed your child? |
| How do you feel about their decisions/the way they do things? |
| Is there anyone else who influences your decision about how much to feed him/her without necessarily being involved in feeding him/her? |
| How confident are you with the people who influence your decisions? |
| **Other influences** |
| Is there any other information that you’ve used to help you with feeding your child? |
| How much have you used this information? Do you trust the information? |
| **Use of general portion size guidance** |
| Do you know of any information or resources that gives guidance/advise on how much to feed your child? |
| **Awareness of and thoughts on existing guidance**  **(Participants shown PowerPoint)** |
| Have you seen any of these before? |
| What are your initial thoughts on seeing these? |
| **Opinions on certain aspects of the guidance** |
| If you were to use this sort of information resource, would you want it to include individual foods and drinks or meal and recipes? |
| How many examples of foods/drinks or meals/recipes do you think is good for a resource to include? |
| Would you rather a resource be age specific, for the exact age of your child (different guidance for 1 years vs 2 years) or more generic, for an age range (1-4 years for example)? |
| Would you want resources to include guidance about foods high in fat and sugar? |
| **Use of the guidance** |
| How would you feel if you were feeding your child more than guidance suggested? |
| After seeing these, would you be interested in using any or looking for this kind or information? |
| In what form would you like the guidance to be in? |
| How would you use/engage with this sort of information? |
| Are there any other things to do with portioning or just feeding in general that you think would be useful to have guidance on? |
